# Cyclic lipopeptide profile of the plant-beneficial endophytic bacterium *Bacillus subtilis* HC8

**DOI:** 10.1007/s00203-012-0823-0

**Published:** 2012-05-31

**Authors:** Natalia Malfanova, Laurent Franzil, Ben Lugtenberg, Vladimir Chebotar, Marc Ongena

**Affiliations:** 1 Sylvius Laboratory, Institute of Biology, Leiden University, Sylviusweg 72, 2333 BE Leiden, The Netherlands; 2 All-Russian Research Institute for Agricultural Microbiology (ARRIAM), Saint-Petersburg-Pushkin, Russia; 3 Walloon Centre for Industrial Biology, Bio-Industry Unit, Gembloux Agro-Bio Tech, University of Liege, 5030 Gembloux, Belgium

**Keywords:** Endophytic, *Bacillus*, Cyclic lipopeptides, LC–MS

## Abstract

In a previous study (Malfanova et al. in Microbial Biotech 4:523–532, 2011), we described the isolation and partial characterization of the biocontrol endophytic bacterium *B. subtilis* HC8. Using thin-layer chromatography, we have detected several bioactive antifungal compounds in the methanolic extract from the acid-precipitated supernatant of HC8. In the present study, we have further analyzed this methanolic extract using liquid chromatography-mass spectrometry. Based on the comparison of retention times and molecular masses with those of known antifungal compounds, we identified three families of lipopeptide antibiotics. These include four iturins A having fatty acyl chain lengths of C14 to C17, eight fengycins A (from C14 to C18 and from C15 to C17 containing a double bond in the acyl chain), four fengycins B (C15 to C18), and five surfactins (C12 to C16). Evaluation of the antifungal activity of the isolated lipopeptides showed that fengycins are the most active ones. To our knowledge, this is the first report of an endophytic *Bacillus subtilis* producing all three major families of lipopeptide antibiotics containing a very heterogeneous mixture of homologues. The questions remain open which of these lipopeptides (1) are being produced during interaction with the plant and (2) are contributing to the biocontrol activity of HC8.

## Introduction

Endophytes are plant-associated microbes that are able to colonize plants internally. Due to the nature of their endophytic lifestyle, they establish a long-lasting stable relationship with a plant. In this symbiotic association, the plant provides nutrients and shelter for the microbes and, in turn, the endophyte can help the plant by protecting it against phytopathogens or by promoting its growth. One of the mechanisms of such a protection includes production of bioactive secondary metabolites which either can be directly involved in antibiosis (Thomashow and Weller 1995; Haas and Défago 2005; Lugtenberg and Kamilova 2009) and/or in triggering induced systemic resistance (ISR) (Tran et al. 2007; Ongena et al. 2007). *Bacillus* spp. are known to produce a wide range of secondary metabolites including cyclic lipopeptides (c-LPs), some of the most powerful ones with regard to their antifungal and biosurfactant activity (Ongena and Jacques 2008; Jacques 2011).

Secondary metabolites produced by *Bacillus* spp. consist mainly of three families of non-ribosomally synthesized c-LPs. These are the iturins, the fengycins, and the surfactins. These c-LPs contain a peptide ring with seven (iturins and surfactins) or 10 (fengycins) amino acids linked to a β-hydroxy (fengycins and surfactins) or β-amino (iturins) fatty acid. Each lipopeptide family is further subdivided into groups based on its amino acid composition. For example, the fengycin family comprises fengycin A and fengycin B, which differ in a single amino acid in the sixth position (d-alanine and d-valine, respectively). Within each group, there are homologues differing in the length, branching, and saturation of their acyl chain (Ongena and Jacques 2008). Members of the iturin family range from C14 to C17, fengycins from C14 to C19, and surfactins from C12 to C17. Both iturins and fengycins are mainly known for their anti-fungal properties, while surfactins are mostly anti-viral and anti-bacterial. When different families are co-produced, their interaction can become synergistic and enhances each of their respective activities (Maget-Dana et al. 1992; Ongena et al. 2007; Romero et al. 2007).

In our previous work (Malfanova et al. 2011), we have described the isolation and partial characterization of the plant-beneficial endophytic bacterium *B. subtilis* HC8. This strain shows strong in vitro antifungal activity against various fungal phytopathogens. When applied to seeds, *B. subtilis* HC8 is able to significantly decrease symptoms of tomato foot and root rot which is caused by the phytopathogen Forl. The crude methanolic extract from the acid-precipitated supernatant fluid of this strain contains several bioactive compounds which behave similar to some known lipopeptide antibiotics on a TLC plate. Taking together, all these data suggested that *B. subtilis* HC8 produces several lipopeptide antibiotics which might be important for its antifungal and biocontrol activities. Therefore, the aims of this study were (1) to identify the putative lipopeptides produced by the beneficial endophytic strain *Bacillus subtilis* HC8, (2) to characterize the antifungal activity of the isolated c-LPs families against Forl in an in vitro bioassay, (3) to test whether there is synergistic activity between the families of c-LPs toward Forl in vitro, and (4) whether active c-LPs affect hyphal morphology.

## Materials and methods

### Extraction of antifungal compounds

The extraction of antifungal compounds was performed as described in our previous study (Malfanova et al. 2011). Briefly, *B. subtilis* HC8 was grown in Brain Heart Infusion broth (BHI, Difco Laboratories, MI, USA) for 60 h at 28 °C. Subsequently, cells were removed by centrifugation at 13,000 r.p.m. for 10 min. The supernatant fluid was acidified to pH 2.0 with concentrated HCl. The resulting precipitate was extracted twice with methanol, the combined extracts were concentrated by vacuum evaporation, and the resulting material was subsequently dissolved in 1/50th of the initial culture volume of methanol.

### Identification of c-LPs using LC–MS analysis

Putative c-LPs were identified as described by Arguelles-Arias et al. (2009) using LC–MS analysis. Briefly, the crude methanolic extract was analyzed by reverse-phase high-pressure liquid chromatography (Waters Alliance 2695/diode array detector) coupled to a quadrupole mass analyzer on an X-Terra MS 150 × 2.1 mm, 3.5 μm C8 column (Waters, Milford, MA, USA). Lipopeptides were eluted using a two component solvent system of which solvent A is water and solvent B is acetonitrile, both acidified with 0.1 % formic acid. We used four different elution programs including one general program to elute all lipopeptides and three family-specific programs to get a better separation and quantification of the different lipopeptides within each family (Table 1). All elution programs used a flow rate of 0.5 ml/min and detection occurred using the positive ion mode.

**Table 1:** Elution programs used in HPLC. Solvent A is water, acidified with 0.1 % formic acid, and solvent B is acetonitrile, acidified with 0.1 % formic acid. The curve indicates the rate at which the solvent is changed to the new compositions, curve 1 is exponential and curve 6 is linear

Time	A%	B%	Curve
a General elution program			
0	57	43	1
1.5	57	43	1
17	37	63	6
17.5	20	80	6
26	0	100	6
27	57	43	6
35	57	43	6
b Iturin-specific elution program			
0	62	38	1
20	55	45	6
25	50	50	6
27	0	100	6
32	0	100	6
33	62	38	6
40	62	38	6
c Fengycin-specific elution program			
0	60	40	1
20	35	65	6
21	0	100	6
26	0	100	6
27	60	40	6
35	60	40	6
d Surfactin-specific elution program			
0	22	78	1
20	22	78	1

Identification of lipopeptides was based on the comparison of retention times and molecular masses with those of known c-LPs (Ongena et al. 2005; Ongena and Jacques 2008). As a control, the 95 % pure authentic standards for each family were used. The fengycin A and B lipopeptides with identical molecular mass and retention time were distinguished as described by Sun et al. (2006) based on the formation of specific product ions upon mild conditions of fragmentation of molecular ions. Product ions with mass-to-charge value (m/z) 966 and 1080 correspond to fengycin A while those at m/z 994 and 1108 correspond to fengycin B. Amount of each lipopeptide family present in the sample was calculated based on calibration curves of purified iturins, fengycins, and surfactins available in the laboratory.

### Evaluation of antifungal activity of the isolated c-LPs

Antifungal activity of the isolated c-LPs was evaluated in the 96-well microtiter plate assay against Forl. To do this, the suspension of fungal spores, adjusted to a density of 5 × 10^5^ spores/ml, was combined either with single compounds dissolved in methanol ranging from 3 to 100 μg/ml or with their combination according to the co-production profile (iturins 47 %, fengycins 36 %, and surfactins 17 %) in a final volume of 150 μl of half strength Potato Dextrose Broth (PDB, Difco Laboratories, MI, USA). In the positive control, c-LPs were replaced with the corresponding volume of methanol. In the negative control, no spores and no c-LPs were added. Inoculated plates were incubated for 25 h at 30 °C and subsequently the fungal growth was determined by measuring the optical density (OD) at 620 nm with a microplate reader. To see the impact of c-LPs on fungal morphology, fungal hyphae treated with 100 μg/ml of c-LPs were observed with an Axioskop2-type microscope using a 40× objective (Carl Zeiss Jena GmbH, Germany). All experiments were performed at least twice.

## Results and discussion

**Fig. 1:**
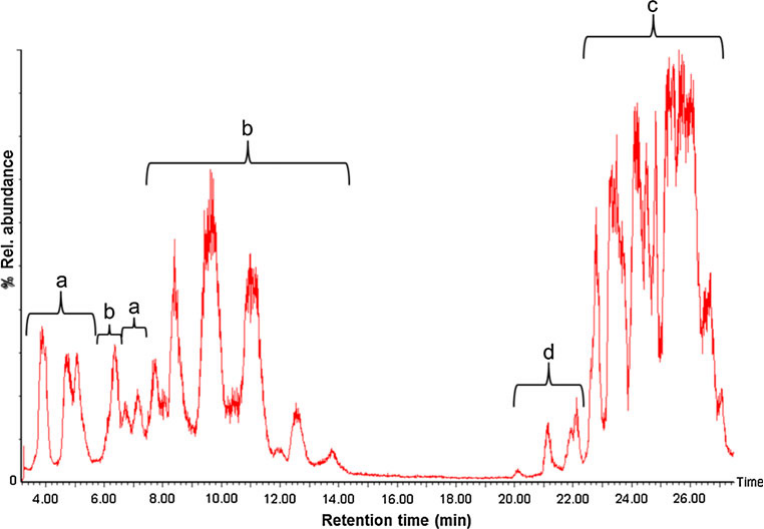
LC–MS analysis of the crude methanolic extract from the acid-precipitated supernatant fluid of *B. subtilis* HC8. Analysis was performed as described by Arguelles-Arias et al. (2009). **a** iturins A; **b** fengycins; **c** surfactins; **d** unknown compounds

LC–MS was performed on a crude methanolic extract of the acid-precipitated supernatant fluid of *B. subtilis* HC8. To elute all putative c-LPs, we used a general elution program (Table 1a). This program uses a gradient of increasing amounts of acetonitrile (a polar solvent) and thus the first eluents include the less polar iturins followed by the increasingly polar fengycins and surfactins (Fig. 1). Iturins and fengycins are less separated compared to surfactins due to the similar polarity of the biggest iturins and the smallest fengycins. Based on calibration curves for standard c-LPs, iturins represent the most abundant family of the lipopeptides (65 μg/ml culture supernatant, followed by fengycins (50 μg/ml) and surfactins (23 μg/ml).

Before the elution of surfactins (20–22 min), several peaks appeared that correspond to unknown compounds that could be related to surfactin lipopeptides based on their chromatographic behavior and fragmentation pattern. Preliminary analysis of their product ions (results not shown) indicates that these compounds contain unusual amino acid(s) in their peptide moiety. The presence of unknown surfactin-like compounds can be either specific for *B. subtilis* HC8 or due to relative abundance of certain amino acids in the medium. A possible influence of the composition of the medium was shown in several studies (Peypoux et al. 1994; Grangemard et al. 1997). For example, addition of l-alanine to the growth medium resulted in incorporation of this amino acid in the fourth position of the peptide ring of surfactins instead of the usual amino acid l-valine (Peypoux et al. 1994). This can be explained by the non-specificity of the adenylation domain of some non-ribosomal peptide synthetases involved in surfactin biosynthesis (Jacques 2011). Additional culturing in various growth media, purification, and analysis would be required to elucidate the exact composition and structure of these minor unknown surfactin-like compounds detected in the present study.

**Fig. 2:**
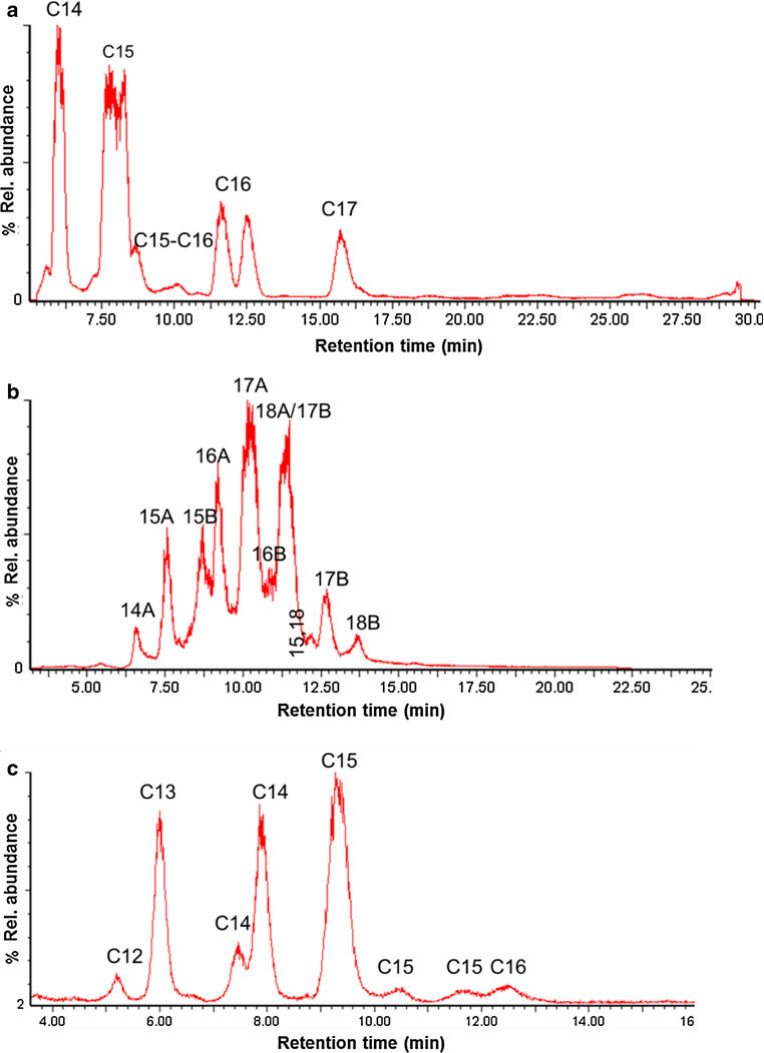
Family-specific LC–MS analysis of the methanolic extract of *B.subtilis* HC8. Each lipopeptide family was characterized using specific elution gradients as described elsewhere (Toure et al. 2004). **a** Iturin-specific analysis. The homologues of iturin A contain C14-C17 acyl chains. **b** Fengycin-specific analysis. A, fengycin A; B, fengycin B; 15, 18 include C15 fengycin A homologue with an unsaturated fatty acyl chain and the saturated C18 homologue. Fengycins A C16 and C17 homologues with a double bond elute together with 18B. **c** Surfactin-specific analysis. The homologues of surfactin contain C12-C16 acyl chains

To obtain a better separation and quantification of the different lipopeptides of the same family, we ran three family-specific programs (Tables 1b–d). The iturin-specific program revealed the presence of all four members of iturin A, having fatty acyl chain lengths from C14 to C17 (Table 1; Fig. 2a). The most abundant homologue is C15, followed by C14, C16, and C17. The fatty acid chain length of iturins is known to be important for their antifungal activity which increases with increasing number of carbon atoms (Bonmatin et al. 2003; Shai et al. 2006; Tabbene et al. 2011). For example, it has been shown that the C16 homologue of bacillomycin D-like compound, which is a member of the iturin family, displayed the strongest fungicidal activity in vitro against *Candida albicans* whereas C14 and C15 homologues showed weak and moderate activity, respectively (Tabbene et al. 2011). This is supposed to be due to the fact that long-chain iturins are more hydrophobic and therefore may interact more effectively with ergosterol-containing membranes of fungi and yeasts. Moreover, Malina and Shai (2005) suggested that the length of the acyl chain can also affect the specificity of lipopeptide–cell membrane interactions. They synthesized several lipopeptides with increasing acyl chain lengths of 10, 12, 14, and 16 carbons to the peptides. Lipopeptides with short fatty acid chains (C10 and C12) displayed both antibacterial and antifungal activity, whereas those with long chains (C14 and C16) were active only against fungi. A possible explanation of this result is that long-chain lipopeptides more readily form oligomers and thus interact easier with the fungal membrane than with the bacterial one (Malina and Shai 2005). This might partly explain the strong antifungal and the limited antibacterial properties of iturins.

**Fig. 3:**
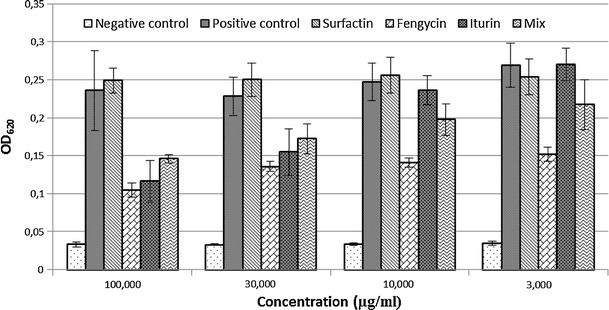
Evaluation of antifungal activity of c-LPs against Forl. The fungal spores were incubated with c-LPs at four different concentrations and with a combination of iturins, fengycins, and surfactins. The inhibition of the fungal growth was judged as the decrease in OD620 compared to the control. *Bars* indicate confidence intervals

**Fig. 4:**
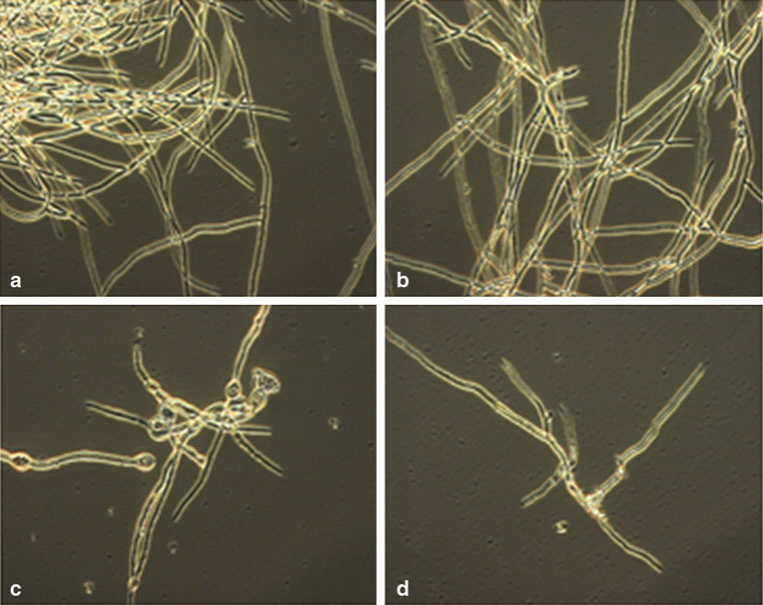
Visualization of the inhibitory effect of c-LPs **a** methanol; **b** surfactins; **c** fengycins; **d** iturins. Fungal spores were treated with a 100 μg/ml of each c-LP and incubated at 30 °C for 25 h

Using the fengycin-specific program, we found eight fengycins A and four fengycins B (Table 2, Fig. 2b). Fengycins A consist of the saturated C14 to C18 homologues and C15 to C17 containing a single double bond in the fatty acyl chain. Fengycins B comprise C15 to C18 homologues with a saturated acyl chain. Fengycins A are present in our sample in a larger quantity compared to fengycins B. The C17 fengycin A is the most abundant homologue while the C15-C17 homologues with unsaturated acyl chain appear to be the least abundant ones. Among fengycins B, C17 is the most abundant homologue and C18 is the least. Although fengycin homologues have long fatty acyl chains (up to C19), they are less hemolytic than iturins and more active toward filamentous fungi (Jacques 2011). Indeed, in our study we found that fengycins are bioactive at all tested concentrations while iturins display an inhibitory effect only at high concentrations (30 and 100 μg/ml) (Fig. 3). Moreover, fengycins alone are significantly more active than the mix of the three c-LPs suggesting that fengycins are the major antifungal compound against Forl. This notion is supported by the microscopic observation of more severe growth restriction of fungal hyphae incubated with fengycins than with any of the other compounds (Fig. 4). The result with the mixture also shows that there is no (strong) synergy in the action of the various c-LPs (Fig. 4).

**Table 2:** c-LPs production by *B. subtilis* HC8 as detected by LC–MS

Cyclic lipopeptide family	Molecular mass [M-H]^+^	Homologue
Iturin A	1043.7	C-14
	1057.74	C-15
	1071.75	C-16
	1085.71	C-17
Fengycin A^a^	1436.18	C-14
	1450.16	C-15
	1464.14	C-16
	1478.12	C-17
	1492.16	C-18
	1448.15	C = 15^c^
	1462.19	C = 16^c^
	1476.1	C = 17^c^
Fengycin B^b^	1478.05	C-15
	1492.16	C-16
	1506.2	C-17
	1521.22	C-18
Surfactin	994.21	C-12
	1008.75	C-13
	1022.33	C-14
	1036.87	C-15
	1050.92	C-16

c-LPs were identified by comparing both their molecular masses and their retention times with those from the literature (Ongena et al. 2005; Ongena and Jacques 2008)

^a^Fengycin A contains the amino acid d-alanine in the sixth position of the peptide ring

^b^Fengycin B contains the amino acid d-valine in the sixth position of the peptide ring

^c^Double bond in the acyl chain

The surfactin-specific program revealed the presence of six out of seven known surfactins with an acyl chain from C12 to C16 and the amino acid leucine at the seventh position of the peptide ring (Table 2; Fig. 2c). The most abundant homologue is C15 followed by C14, C13, C12, and C16. Numerous studies showed that C14 and C15 surfactin homologues are the most bioactive ones with respect to their antiviral activity (Kracht et al. 1999), insecticidal activity (Assie et al. 2002), triggering several plant-defense mechanisms (Jourdan et al. 2009), and foaming properties (Razafindralambo et al. 1998). Although surfactins do not show significant antifungal activity at the concentrations tested (see Figs. 3,4), they can favor establishment and spreading of biocontrol bacteria in internal host tissues. LP has been shown to be implicated in a flagella-independent surface motility (Kinsinger et al. 2003; Leclère et al. 2006) and in the formation of biofilms (Hofemeister et al. 2004) thereby globally contributing to the ability of some bacilli to efficiently colonize surfaces of plant roots (Bais et al. 2004).

In this study, we show for the first time that an endophytic *B. subtilis* strain is able to produce all three families of c-LPs of which the fengycins displayed the strongest antifungal activity against Forl. Production of fengycins A and B was also reported for the endophytic bacteria *B. amyloliquefaciens* ES-2 (Sun et al. 2006) and *B. subtilis* B-FS01 (Hu et al. 2007). However, in contrast to HC8, neither of these strains co-produces significant amounts of surfactin, fengycin, and iturin. Moreover, a very heterogeneous mixture of homologues was detected in the methanolic extract of our strain. Whether the same c-LPs and homologues are being produced during interaction of *B. subtilis* HC8 with plants and which of them are involved in its biocontrol activity remains to be established.
